# 4-Nitro­phenyl 2-methyl­benzoate

**DOI:** 10.1107/S1600536809046005

**Published:** 2009-11-07

**Authors:** Uzma Bibi, Humaira M. Siddiqi, Michael Bolte, Zareen Akhter

**Affiliations:** a Department of Chemistry, Quaid-I-Azam University, Islamabad 45320, Pakistan; b Institut für Anorganische Chemie, J.-W.-Goethe-Universität Frankfurt, Max-von-Laue-Strasse 7, 60438 Frankfurt/Main, Germany

## Abstract

The title compound, C_14_H_11_NO_4_, crystallizes with two mol­ecules in the asymmetric unit. The major conformational difference between these two mol­ecules is the dihedral angle between the aromatic rings, namely 36.99 (5) and 55.04 (5)°. The nitro groups are coplanar with the phenyl rings to which they are attached, the O—N—C—C torsion angles being −1.9 (3) and 1.0 (3)° in the two mol­ecules.

## Related literature

For background to the applications of aromatic esters containing nitro groups in their aromatic rings, see: Jefford & Zaslona (1985 ▶); Jefford *et al.* (1986 ▶); Schauble *et al.* (1971 ▶). For related structures, see: Adams & Morsi (1976 ▶); Shibakami & Sekiya (1995 ▶).
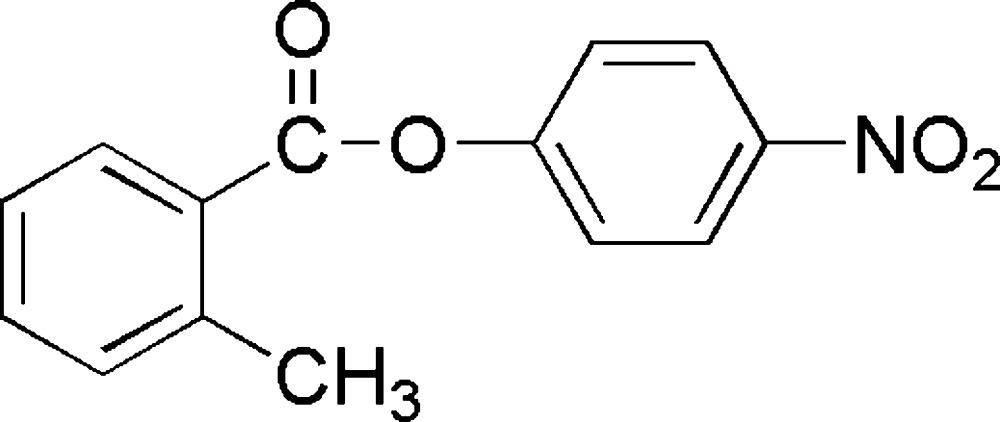


## Experimental

### 

#### Crystal data

C_14_H_11_NO_4_
*M* *_r_* = 257.24
Orthorhombic, 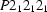

*a* = 11.4748 (7) Å
*b* = 14.3608 (8) Å
*c* = 14.5944 (9) Å
*V* = 2405.0 (2) Å^3^
*Z* = 8
Mo *K*α radiation
μ = 0.11 mm^−1^
*T* = 173 K
0.48 × 0.43 × 0.42 mm

#### Data collection

Stoe IPDS II two-circle diffractometer
Absorption correction: none
8396 measured reflections
2536 independent reflections
2233 reflections with *I* > 2σ(*I*)
*R*_int_ = 0.032

#### Refinement

*R*[*F* ^2^ > 2σ(*F* ^2^)] = 0.032
*wR*(*F* ^2^) = 0.081
*S* = 1.00
2536 reflections
346 parameters
H-atom parameters constrained
Δρ_max_ = 0.19 e Å^−3^
Δρ_min_ = −0.16 e Å^−3^

### 

Data collection: *X-AREA* (Stoe & Cie, 2001 ▶); cell refinement: *X-AREA*; data reduction: *X-AREA*; program(s) used to solve structure: *SHELXS97* (Sheldrick, 2008 ▶); program(s) used to refine structure: *SHELXL97* (Sheldrick, 2008 ▶); molecular graphics: *XP* in *SHELXTL* (Sheldrick, 2008 ▶); software used to prepare material for publication: *SHELXL97*.

## Supplementary Material

Crystal structure: contains datablocks I, global. DOI: 10.1107/S1600536809046005/pv2221sup1.cif

Structure factors: contains datablocks I. DOI: 10.1107/S1600536809046005/pv2221Isup2.hkl

Additional supplementary materials:  crystallographic information; 3D view; checkCIF report

## Figures and Tables

**Fig. 1.**
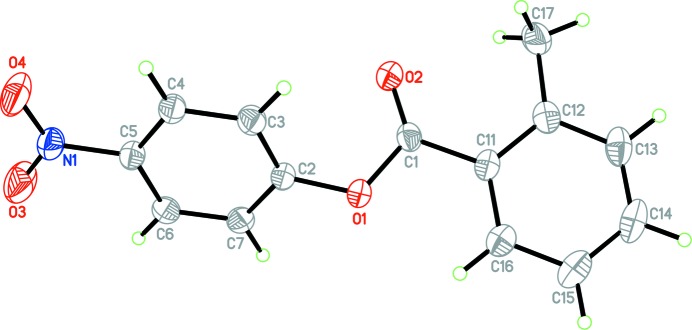
Molecular structure of the independent molecule of the title compound with displacement parameters drawn at the 50% probability level.

**Fig. 2.**
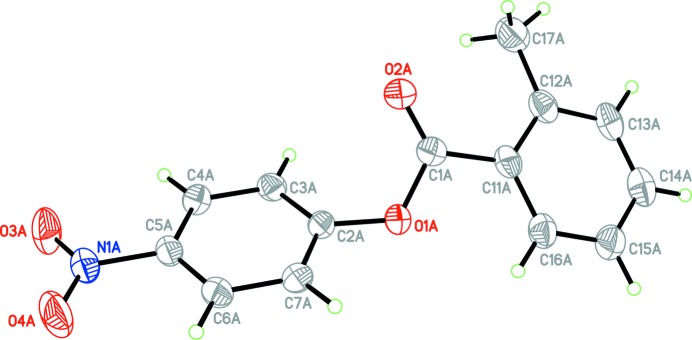
Molecular structure of the other independent molecule of the title compound with displacement parameters drawn at the 50% probability level.

## supplementary crystallographic information

### Comment

Aromatic esters containing nitro groups in their aromatic rings are potential precursors for the preparation of compounds with a number of biological activities such as analgesic and anti-inflammatory (Jefford & Zaslona, 1985). In addition, these compounds served as potential intermediates in the synthesis of many natural products (Jefford *et al.,* 1986; Schauble *et al.*, 1971). The nitro group can be reduced to amino group which can be utilized for the synthesis of azoxy compounds. We have synthsized the title compound (I) which is a nitro substituted ester. In this article, the crystal structure of (I) is reported.

The title compound crystallizes with two molecules (Fig. 1) in an asymmetric unit. The major conformational difference between the two molecules is the dihedral angle between the aromatic rings, namely 36.99 (5)° and 55.04 (5)°. The nitro groups in both molecules are coplanar with the phenyl rings to which they are attached with dihedral angles O3—N1—C5—C6 and O3A—N1A—C5A—C4A being -1.9 (3) and 1.0 (3)°, respectively. The bond distances and angles in (I) agree well with the corresponding distances and angles reported in closely related structures (Adams & Morsi, 1976; Shibakami & Sekiya, 1995).

### Experimental

2-Toluic acid (1.5 g, 1 mol) in a 100 ml two neck round bottom flask was gradually warmed on a water bath to 323 K. Dry thionyl chloride was added in excess slowly with stirring along with 2–3 drops of DMF as catalyst. The mixture was refluxed for about 50–60 minutes at 343 K. The excess of thionyl chloride was removed by repeated evaporation at reduced pressure. In a separate flask, 4-nitrophenol (1.5 g, 0.0065 mol) was dissolved in dry dichloromethane to which triethyl amine was added at room temperature to get transparent solution. The acid chloride was added into it drop wise with constant stirring at room temperature for 30 minutes under anhydrous condition and then poured into 20 ml of cold water. Excess of triethyl amine was removed by adding cold dilute HCl solution. The reaction was monitored by TLC using ethyl acetate: n-hexane (1:2). After the completion of reaction the oily product was allowed to settle down and the supernatant liquid was decanted. The product was stirred well with distilled water and extracted with ethyl acetate (3 x 40 ml), washed with 5% NaHCO_3_ solution and dried over anhydrous Na_2_SO_4_. After filtration the solution was concentrated to obtain the title compound which was recrystallized from n-hexane (Yield: 37 %; m.p. 336-344 K).

### Refinement

H atoms were positioned geometrically and refined using a riding model with with C—H distances 0.95 and 0.98 Å for aromatic and methyl H-atoms, respectively, and displacement parameters, *U_iso_* = 1.2 and 1.5 times *U_eq_* of aromatic and methyl C-atoms, respectively. The methyl groups were allowed to rotate but not to tip. Due to the absence of anomalous scatterers, the absolute structure could not be determined which was set arbitrarily and Friedel pairs (1929) were merged.

### Crystal data

**Table:**

C_14_H_11_NO_4_	*F*(000) = 1072
*M**_r_* = 257.24	*D*_x_ = 1.421 Mg m^−^^3^
Orthorhombic, *P*2_1_2_1_2_1_	Mo *K*α radiation, λ = 0.71073 Å
Hall symbol: P 2ac 2ab	Cell parameters from 7991 reflections
*a* = 11.4748 (7) Å	θ = 3.4–25.9°
*b* = 14.3608 (8) Å	µ = 0.11 mm^−^^1^
*c* = 14.5944 (9) Å	*T* = 173 K
*V* = 2405.0 (2) Å^3^	Block, colourless
*Z* = 8	0.48 × 0.43 × 0.42 mm

### Data collection

**Table:**

Stoe IPDS II two-circle diffractometer	2233 reflections with *I* > 2σ(*I*)
Radiation source: fine-focus sealed tube	*R*_int_ = 0.032
graphite	θ_max_ = 25.6°, θ_min_ = 3.4°
ω scans	*h* = −13→11
8396 measured reflections	*k* = −17→16
2536 independent reflections	*l* = −17→15

### Refinement

**Table:**

Refinement on *F*^2^	Secondary atom site location: difference Fourier map
Least-squares matrix: full	Hydrogen site location: inferred from neighbouring sites
*R*[*F*^2^ > 2σ(*F*^2^)] = 0.032	H-atom parameters constrained
*wR*(*F*^2^) = 0.081	*w* = 1/[σ^2^(*F*_o_^2^) + (0.0582*P*)^2^] where *P* = (*F*_o_^2^ + 2*F*_c_^2^)/3
*S* = 1.00	(Δ/σ)_max_ < 0.001
2536 reflections	Δρ_max_ = 0.19 e Å^−^^3^
346 parameters	Δρ_min_ = −0.16 e Å^−^^3^
0 restraints	Extinction correction: *SHELXL97* (Sheldrick, 2008), Fc^*^=kFc[1+0.001xFc^2^λ^3^/sin(2θ)]^-1/4^
Primary atom site location: structure-invariant direct methods	Extinction coefficient: 0.0029 (6)

### Special details

**Table:**

Geometry. All e.s.d.'s (except the e.s.d. in the dihedral angle between two l.s. planes) are estimated using the full covariance matrix. The cell e.s.d.'s are taken into account individually in the estimation of e.s.d.'s in distances, angles and torsion angles; correlations between e.s.d.'s in cell parameters are only used when they are defined by crystal symmetry. An approximate (isotropic) treatment of cell e.s.d.'s is used for estimating e.s.d.'s involving l.s. planes.
Refinement. Refinement of *F*^2^ against ALL reflections. The weighted *R*-factor *wR* and goodness of fit *S* are based on *F*^2^, conventional *R*-factors *R* are based on *F*, with *F* set to zero for negative *F*^2^. The threshold expression of *F*^2^ > σ(*F*^2^) is used only for calculating *R*-factors(gt) *etc*. and is not relevant to the choice of reflections for refinement. *R*-factors based on *F*^2^ are statistically about twice as large as those based on *F*, and *R*- factors based on ALL data will be even larger.

### Fractional atomic coordinates and isotropic or equivalent isotropic displacement parameters (Å^2^)

**Table:**

	*x*	*y*	*z*	*U*_iso_*/*U*_eq_	
O1	0.16002 (13)	0.61145 (10)	0.13451 (12)	0.0313 (4)	
O2	−0.01252 (14)	0.62719 (11)	0.06228 (12)	0.0350 (4)	
O3	0.20623 (17)	0.17957 (12)	0.15433 (18)	0.0603 (6)	
O4	0.04224 (17)	0.19478 (12)	0.22316 (14)	0.0484 (5)	
N1	0.12619 (18)	0.22748 (13)	0.18336 (14)	0.0330 (5)	
C1	0.07279 (19)	0.66283 (14)	0.09413 (15)	0.0252 (4)	
C2	0.14470 (18)	0.51564 (14)	0.14478 (14)	0.0257 (5)	
C3	0.0478 (2)	0.47887 (15)	0.18780 (15)	0.0279 (5)	
H3	−0.0134	0.5184	0.2081	0.033*	
C4	0.04139 (19)	0.38314 (15)	0.20091 (15)	0.0269 (5)	
H4	−0.0242	0.3559	0.2303	0.032*	
C5	0.13290 (19)	0.32834 (15)	0.17014 (15)	0.0262 (5)	
C6	0.23122 (19)	0.36492 (15)	0.12783 (15)	0.0276 (5)	
H6	0.2929	0.3257	0.1080	0.033*	
C7	0.23633 (19)	0.46020 (15)	0.11547 (15)	0.0276 (5)	
H7	0.3024	0.4876	0.0870	0.033*	
C11	0.10142 (19)	0.76401 (15)	0.09643 (15)	0.0257 (4)	
C12	0.0246 (2)	0.82974 (15)	0.05781 (15)	0.0289 (5)	
C13	0.0559 (2)	0.92345 (16)	0.06402 (16)	0.0356 (5)	
H13	0.0054	0.9692	0.0389	0.043*	
C14	0.1581 (3)	0.95161 (16)	0.10555 (17)	0.0398 (6)	
H14	0.1767	1.0160	0.1088	0.048*	
C15	0.2334 (2)	0.88691 (17)	0.14233 (17)	0.0389 (6)	
H15	0.3043	0.9062	0.1701	0.047*	
C16	0.2045 (2)	0.79333 (16)	0.13842 (16)	0.0319 (5)	
H16	0.2554	0.7485	0.1646	0.038*	
C17	−0.0882 (2)	0.80479 (18)	0.01122 (19)	0.0397 (6)	
H17A	−0.1438	0.7815	0.0568	0.059*	
H17B	−0.0738	0.7564	−0.0348	0.059*	
H17C	−0.1205	0.8602	−0.0187	0.059*	
O1A	0.17914 (14)	0.15192 (10)	0.86948 (12)	0.0341 (4)	
O2A	0.02001 (17)	0.18630 (13)	0.95339 (14)	0.0502 (5)	
O3A	−0.00192 (18)	−0.24935 (12)	0.77876 (14)	0.0505 (5)	
O4A	0.16863 (19)	−0.28107 (13)	0.83276 (17)	0.0582 (6)	
N1A	0.09144 (18)	−0.22567 (13)	0.81235 (14)	0.0338 (5)	
C1A	0.1056 (2)	0.21228 (16)	0.91421 (16)	0.0323 (5)	
C2A	0.15008 (19)	0.05831 (15)	0.86021 (15)	0.0275 (5)	
C3A	0.0438 (2)	0.03078 (15)	0.82310 (16)	0.0290 (5)	
H3A	−0.0143	0.0755	0.8086	0.035*	
C4A	0.0245 (2)	−0.06321 (15)	0.80779 (14)	0.0290 (5)	
H4A	−0.0475	−0.0842	0.7831	0.035*	
C5A	0.1119 (2)	−0.12599 (15)	0.82913 (15)	0.0273 (5)	
C6A	0.2179 (2)	−0.09892 (16)	0.86513 (16)	0.0309 (5)	
H6A	0.2764	−0.1436	0.8788	0.037*	
C7A	0.23671 (19)	−0.00483 (17)	0.88083 (16)	0.0301 (5)	
H7A	0.3087	0.0159	0.9056	0.036*	
C11A	0.1498 (2)	0.30931 (16)	0.90502 (15)	0.0314 (5)	
C12A	0.0759 (2)	0.38603 (17)	0.91703 (16)	0.0358 (5)	
C13A	0.1220 (2)	0.47552 (17)	0.90166 (17)	0.0393 (6)	
H13A	0.0727	0.5283	0.9076	0.047*	
C14A	0.2374 (3)	0.48793 (17)	0.87809 (18)	0.0417 (6)	
H14A	0.2664	0.5490	0.8680	0.050*	
C15A	0.3114 (2)	0.41263 (18)	0.86902 (18)	0.0418 (6)	
H15A	0.3913	0.4218	0.8545	0.050*	
C16A	0.2678 (2)	0.32367 (16)	0.88133 (16)	0.0362 (5)	
H16A	0.3179	0.2716	0.8738	0.043*	
C17A	−0.0493 (2)	0.3775 (2)	0.9429 (2)	0.0478 (7)	
H17D	−0.0891	0.3366	0.8992	0.072*	
H17E	−0.0858	0.4392	0.9418	0.072*	
H17F	−0.0555	0.3512	1.0046	0.072*	

### Atomic displacement parameters (Å^2^)

**Table:**

	*U*^11^	*U*^22^	*U*^33^	*U*^12^	*U*^13^	*U*^23^
O1	0.0282 (8)	0.0198 (7)	0.0458 (9)	−0.0024 (6)	−0.0051 (7)	0.0052 (7)
O2	0.0326 (9)	0.0235 (7)	0.0490 (10)	−0.0024 (7)	−0.0090 (7)	−0.0003 (7)
O3	0.0469 (11)	0.0246 (9)	0.1094 (18)	0.0035 (9)	0.0207 (12)	−0.0050 (10)
O4	0.0533 (12)	0.0290 (9)	0.0628 (12)	−0.0075 (9)	0.0194 (10)	0.0070 (8)
N1	0.0342 (11)	0.0216 (9)	0.0431 (11)	−0.0006 (9)	0.0008 (9)	−0.0007 (8)
C1	0.0267 (11)	0.0211 (10)	0.0277 (10)	0.0031 (9)	0.0032 (9)	0.0001 (9)
C2	0.0270 (11)	0.0202 (10)	0.0299 (11)	−0.0019 (9)	−0.0037 (9)	0.0019 (8)
C3	0.0263 (11)	0.0256 (11)	0.0318 (12)	0.0024 (9)	0.0033 (9)	−0.0006 (8)
C4	0.0267 (11)	0.0235 (10)	0.0305 (11)	−0.0003 (9)	0.0020 (9)	0.0016 (8)
C5	0.0285 (11)	0.0213 (10)	0.0289 (10)	−0.0010 (9)	−0.0008 (9)	0.0006 (8)
C6	0.0240 (10)	0.0279 (11)	0.0310 (11)	0.0027 (9)	0.0012 (9)	−0.0005 (9)
C7	0.0229 (10)	0.0285 (11)	0.0313 (11)	−0.0021 (9)	0.0014 (9)	0.0042 (9)
C11	0.0305 (11)	0.0210 (10)	0.0256 (10)	−0.0011 (9)	0.0058 (9)	−0.0006 (8)
C12	0.0322 (12)	0.0264 (11)	0.0280 (11)	0.0026 (9)	0.0084 (9)	0.0028 (8)
C13	0.0474 (14)	0.0240 (11)	0.0353 (13)	0.0028 (11)	0.0123 (11)	0.0033 (9)
C14	0.0614 (17)	0.0225 (11)	0.0356 (12)	−0.0074 (11)	0.0124 (12)	−0.0028 (10)
C15	0.0490 (15)	0.0339 (13)	0.0339 (12)	−0.0134 (12)	0.0030 (11)	−0.0048 (10)
C16	0.0349 (12)	0.0284 (11)	0.0326 (12)	−0.0034 (10)	0.0008 (10)	−0.0029 (9)
C17	0.0353 (13)	0.0329 (13)	0.0508 (15)	0.0036 (11)	−0.0013 (11)	0.0137 (11)
O1A	0.0339 (8)	0.0227 (8)	0.0458 (9)	−0.0038 (7)	0.0081 (8)	−0.0041 (7)
O2A	0.0481 (11)	0.0414 (10)	0.0612 (12)	−0.0108 (9)	0.0244 (10)	−0.0146 (9)
O3A	0.0573 (12)	0.0319 (9)	0.0624 (13)	−0.0084 (9)	−0.0172 (10)	−0.0089 (8)
O4A	0.0563 (12)	0.0258 (9)	0.0926 (16)	0.0109 (9)	−0.0109 (11)	−0.0031 (10)
N1A	0.0404 (11)	0.0262 (10)	0.0349 (11)	−0.0008 (9)	−0.0004 (9)	−0.0034 (8)
C1A	0.0328 (12)	0.0315 (12)	0.0326 (12)	0.0001 (10)	0.0041 (10)	−0.0022 (10)
C2A	0.0303 (11)	0.0239 (10)	0.0283 (10)	−0.0027 (9)	0.0064 (10)	0.0003 (9)
C3A	0.0275 (11)	0.0262 (10)	0.0332 (11)	0.0046 (9)	0.0006 (10)	0.0028 (9)
C4A	0.0290 (11)	0.0294 (11)	0.0285 (11)	−0.0016 (10)	−0.0027 (9)	0.0014 (9)
C5A	0.0318 (11)	0.0234 (10)	0.0267 (10)	−0.0005 (9)	0.0010 (9)	−0.0014 (8)
C6A	0.0290 (11)	0.0276 (11)	0.0361 (12)	0.0047 (9)	−0.0004 (10)	0.0001 (10)
C7A	0.0242 (10)	0.0320 (11)	0.0343 (12)	−0.0011 (9)	−0.0011 (9)	−0.0017 (9)
C11A	0.0400 (13)	0.0285 (11)	0.0257 (10)	−0.0009 (10)	−0.0022 (10)	−0.0048 (9)
C12A	0.0410 (13)	0.0358 (13)	0.0307 (12)	0.0030 (11)	−0.0060 (10)	−0.0087 (10)
C13A	0.0528 (15)	0.0317 (12)	0.0334 (12)	0.0020 (11)	−0.0113 (12)	−0.0079 (10)
C14A	0.0594 (16)	0.0283 (13)	0.0374 (13)	−0.0031 (12)	−0.0043 (12)	−0.0024 (10)
C15A	0.0449 (14)	0.0360 (13)	0.0446 (14)	−0.0087 (12)	0.0046 (12)	−0.0023 (11)
C16A	0.0451 (14)	0.0268 (12)	0.0366 (13)	−0.0042 (11)	0.0013 (11)	−0.0005 (9)
C17A	0.0438 (15)	0.0465 (15)	0.0532 (16)	0.0061 (13)	−0.0041 (13)	−0.0124 (12)

### Geometric parameters (Å, °)

**Table:**

O1—C1	1.376 (3)	O1A—C1A	1.375 (3)
O1—C2	1.395 (2)	O1A—C2A	1.392 (3)
O2—C1	1.199 (3)	O2A—C1A	1.196 (3)
O3—N1	1.223 (3)	O3A—N1A	1.226 (3)
O4—N1	1.219 (3)	O4A—N1A	1.227 (3)
N1—C5	1.463 (3)	N1A—C5A	1.471 (3)
C1—C11	1.490 (3)	C1A—C11A	1.489 (3)
C2—C3	1.382 (3)	C2A—C7A	1.379 (3)
C2—C7	1.387 (3)	C2A—C3A	1.392 (3)
C3—C4	1.390 (3)	C3A—C4A	1.386 (3)
C3—H3	0.9500	C3A—H3A	0.9500
C4—C5	1.387 (3)	C4A—C5A	1.384 (3)
C4—H4	0.9500	C4A—H4A	0.9500
C5—C6	1.389 (3)	C5A—C6A	1.381 (3)
C6—C7	1.381 (3)	C6A—C7A	1.387 (3)
C6—H6	0.9500	C6A—H6A	0.9500
C7—H7	0.9500	C7A—H7A	0.9500
C11—C16	1.397 (3)	C11A—C12A	1.401 (3)
C11—C12	1.409 (3)	C11A—C16A	1.412 (4)
C12—C13	1.396 (3)	C12A—C13A	1.408 (4)
C12—C17	1.505 (3)	C12A—C17A	1.491 (4)
C13—C14	1.381 (4)	C13A—C14A	1.380 (4)
C13—H13	0.9500	C13A—H13A	0.9500
C14—C15	1.377 (4)	C14A—C15A	1.381 (4)
C14—H14	0.9500	C14A—H14A	0.9500
C15—C16	1.385 (3)	C15A—C16A	1.384 (4)
C15—H15	0.9500	C15A—H15A	0.9500
C16—H16	0.9500	C16A—H16A	0.9500
C17—H17A	0.9800	C17A—H17D	0.9800
C17—H17B	0.9800	C17A—H17E	0.9800
C17—H17C	0.9800	C17A—H17F	0.9800
			
C1—O1—C2	118.90 (16)	C1A—O1A—C2A	120.55 (18)
O4—N1—O3	122.80 (19)	O3A—N1A—O4A	123.2 (2)
O4—N1—C5	119.06 (19)	O3A—N1A—C5A	118.4 (2)
O3—N1—C5	118.1 (2)	O4A—N1A—C5A	118.4 (2)
O2—C1—O1	122.09 (18)	O2A—C1A—O1A	122.3 (2)
O2—C1—C11	127.2 (2)	O2A—C1A—C11A	127.9 (2)
O1—C1—C11	110.67 (18)	O1A—C1A—C11A	109.76 (19)
C3—C2—C7	122.06 (19)	C7A—C2A—C3A	122.0 (2)
C3—C2—O1	121.81 (19)	C7A—C2A—O1A	116.19 (19)
C7—C2—O1	115.96 (19)	C3A—C2A—O1A	121.5 (2)
C2—C3—C4	118.9 (2)	C4A—C3A—C2A	118.7 (2)
C2—C3—H3	120.5	C4A—C3A—H3A	120.7
C4—C3—H3	120.5	C2A—C3A—H3A	120.7
C5—C4—C3	118.5 (2)	C5A—C4A—C3A	118.8 (2)
C5—C4—H4	120.8	C5A—C4A—H4A	120.6
C3—C4—H4	120.8	C3A—C4A—H4A	120.6
C4—C5—C6	123.0 (2)	C6A—C5A—C4A	122.7 (2)
C4—C5—N1	118.6 (2)	C6A—C5A—N1A	118.5 (2)
C6—C5—N1	118.4 (2)	C4A—C5A—N1A	118.7 (2)
C7—C6—C5	117.8 (2)	C5A—C6A—C7A	118.3 (2)
C7—C6—H6	121.1	C5A—C6A—H6A	120.8
C5—C6—H6	121.1	C7A—C6A—H6A	120.8
C6—C7—C2	119.8 (2)	C2A—C7A—C6A	119.5 (2)
C6—C7—H7	120.1	C2A—C7A—H7A	120.3
C2—C7—H7	120.1	C6A—C7A—H7A	120.3
C16—C11—C12	120.2 (2)	C12A—C11A—C16A	119.7 (2)
C16—C11—C1	119.4 (2)	C12A—C11A—C1A	121.2 (2)
C12—C11—C1	120.4 (2)	C16A—C11A—C1A	119.0 (2)
C13—C12—C11	117.3 (2)	C11A—C12A—C13A	118.1 (2)
C13—C12—C17	118.7 (2)	C11A—C12A—C17A	123.4 (2)
C11—C12—C17	124.0 (2)	C13A—C12A—C17A	118.5 (2)
C14—C13—C12	122.0 (2)	C14A—C13A—C12A	121.2 (2)
C14—C13—H13	119.0	C14A—C13A—H13A	119.4
C12—C13—H13	119.0	C12A—C13A—H13A	119.4
C15—C14—C13	120.4 (2)	C13A—C14A—C15A	120.8 (2)
C15—C14—H14	119.8	C13A—C14A—H14A	119.6
C13—C14—H14	119.8	C15A—C14A—H14A	119.6
C14—C15—C16	119.2 (2)	C14A—C15A—C16A	119.2 (2)
C14—C15—H15	120.4	C14A—C15A—H15A	120.4
C16—C15—H15	120.4	C16A—C15A—H15A	120.4
C15—C16—C11	120.9 (2)	C15A—C16A—C11A	120.9 (2)
C15—C16—H16	119.6	C15A—C16A—H16A	119.6
C11—C16—H16	119.6	C11A—C16A—H16A	119.6
C12—C17—H17A	109.5	C12A—C17A—H17D	109.5
C12—C17—H17B	109.5	C12A—C17A—H17E	109.5
H17A—C17—H17B	109.5	H17D—C17A—H17E	109.5
C12—C17—H17C	109.5	C12A—C17A—H17F	109.5
H17A—C17—H17C	109.5	H17D—C17A—H17F	109.5
H17B—C17—H17C	109.5	H17E—C17A—H17F	109.5
			
C2—O1—C1—O2	−4.9 (3)	C2A—O1A—C1A—O2A	6.6 (4)
C2—O1—C1—C11	175.37 (18)	C2A—O1A—C1A—C11A	−173.3 (2)
C1—O1—C2—C3	−53.7 (3)	C1A—O1A—C2A—C7A	−133.2 (2)
C1—O1—C2—C7	130.9 (2)	C1A—O1A—C2A—C3A	53.3 (3)
C7—C2—C3—C4	−0.9 (3)	C7A—C2A—C3A—C4A	0.9 (3)
O1—C2—C3—C4	−176.0 (2)	O1A—C2A—C3A—C4A	174.1 (2)
C2—C3—C4—C5	0.0 (3)	C2A—C3A—C4A—C5A	−0.6 (3)
C3—C4—C5—C6	0.8 (3)	C3A—C4A—C5A—C6A	0.0 (3)
C3—C4—C5—N1	−179.7 (2)	C3A—C4A—C5A—N1A	−179.7 (2)
O4—N1—C5—C4	−2.0 (3)	O3A—N1A—C5A—C6A	−178.6 (2)
O3—N1—C5—C4	178.5 (2)	O4A—N1A—C5A—C6A	1.4 (3)
O4—N1—C5—C6	177.5 (2)	O3A—N1A—C5A—C4A	1.0 (3)
O3—N1—C5—C6	−1.9 (3)	O4A—N1A—C5A—C4A	−178.9 (2)
C4—C5—C6—C7	−0.7 (3)	C4A—C5A—C6A—C7A	0.3 (3)
N1—C5—C6—C7	179.8 (2)	N1A—C5A—C6A—C7A	180.0 (2)
C5—C6—C7—C2	−0.2 (3)	C3A—C2A—C7A—C6A	−0.6 (4)
C3—C2—C7—C6	1.0 (3)	O1A—C2A—C7A—C6A	−174.1 (2)
O1—C2—C7—C6	176.4 (2)	C5A—C6A—C7A—C2A	−0.1 (4)
O2—C1—C11—C16	178.7 (2)	O2A—C1A—C11A—C12A	−20.9 (4)
O1—C1—C11—C16	−1.6 (3)	O1A—C1A—C11A—C12A	159.0 (2)
O2—C1—C11—C12	−0.4 (4)	O2A—C1A—C11A—C16A	160.8 (3)
O1—C1—C11—C12	179.30 (19)	O1A—C1A—C11A—C16A	−19.3 (3)
C16—C11—C12—C13	−0.3 (3)	C16A—C11A—C12A—C13A	2.2 (3)
C1—C11—C12—C13	178.79 (19)	C1A—C11A—C12A—C13A	−176.1 (2)
C16—C11—C12—C17	180.0 (2)	C16A—C11A—C12A—C17A	−179.4 (2)
C1—C11—C12—C17	−0.9 (3)	C1A—C11A—C12A—C17A	2.3 (4)
C11—C12—C13—C14	0.4 (3)	C11A—C12A—C13A—C14A	−1.9 (4)
C17—C12—C13—C14	−179.8 (2)	C17A—C12A—C13A—C14A	179.6 (2)
C12—C13—C14—C15	0.2 (4)	C12A—C13A—C14A—C15A	−0.1 (4)
C13—C14—C15—C16	−1.0 (4)	C13A—C14A—C15A—C16A	1.8 (4)
C14—C15—C16—C11	1.1 (4)	C14A—C15A—C16A—C11A	−1.5 (4)
C12—C11—C16—C15	−0.4 (3)	C12A—C11A—C16A—C15A	−0.5 (4)
C1—C11—C16—C15	−179.6 (2)	C1A—C11A—C16A—C15A	177.8 (2)
